# Loss of the MAF Transcription Factor in Laryngeal Squamous Cell Carcinoma

**DOI:** 10.3390/biom11071035

**Published:** 2021-07-15

**Authors:** Joanna Janiszewska, Magdalena Bodnar, Julia Paczkowska, Adam Ustaszewski, Maciej J. Smialek, Lukasz Szylberg, Andrzej Marszalek, Katarzyna Kiwerska, Reidar Grenman, Krzysztof Szyfter, Malgorzata Wierzbicka, Maciej Giefing, Malgorzata Jarmuz-Szymczak

**Affiliations:** 1 Institute of Human Genetics, Polish Academy of Sciences, 60-479 Poznan, Poland; julia.paczkowska@igcz.poznan.pl (J.P.); adam.ustaszewski@igcz.poznan.pl (A.U.); maciej.smialek@igcz.poznan.pl (M.J.S.); katarzyna.kiwerska@igcz.poznan.pl (K.K.); krzysztof.szyfter@igcz.poznan.pl (K.S.); otosk2@ump.edu.pl (M.W.); maciej.giefing@igcz.poznan.pl (M.G.); malgorzata.jarmuz-szymczak@igcz.poznan.pl (M.J.-S.); 2 Department of Clinical Pathomorphology, Collegium Medicum in Bydgoszcz, Nicolaus Copernicus University in Torun, 85-094 Bydgoszcz, Poland; magdabodnar@o2.pl (M.B.); l.szylberg@gmail.com (L.S.); 3 Department of Oncologic Pathology, Cancer Center, 85-796 Bydgoszcz, Poland; 4 Department of Oncologic Pathology and Prophylactics, Poznan University of Medical Sciences and Greater Poland Cancer Center, 61-866 Poznan, Poland; amars@ump.edu.pl; 5 Department of Tumor Pathology, Greater Poland Cancer Centre, 61-866 Poznan, Poland; 6 Department of Otorhinolaryngology-Head and Neck Surgery, Turku University and Turku University Hospital, 20-500 Turku, Finland; seigre@utu.fi; 7 Department of Otolaryngology and Laryngological Oncology, Poznan University of Medical Sciences, 60-355 Poznan, Poland; 8 Department of Hematology and Bone Marrow Transplantation, Poznan University of Medical Sciences, 61-569 Poznan, Poland

**Keywords:** laryngeal squamous cell carcinoma, microRNAs, transcription factor, MAF, miR-1290, apoptosis

## Abstract

MAF is a transcription factor that may act either as a tumor suppressor or as an oncogene, depending on cell type. We have shown previously that the overexpressed miR-1290 influences MAF protein levels in LSCC (laryngeal squamous cell carcinoma) cell lines. In this study, we shed further light on the interaction between miR-1290 and *MAF*, as well as on cellular MAF protein localization in LSCC. We confirmed the direct interaction between miR-1290 and *MAF* 3′UTR by a dual-luciferase reporter assay. In addition, we used immunohistochemistry staining to analyze MAF protein distribution and observed loss of MAF nuclear expression in 58% LSCC samples, of which 10% showed complete absence of MAF, compared to nuclear and cytoplasmatic expression in 100% normal mucosa. Using TCGA data, bisulfite pyrosequencing and CNV analysis, we excluded the possibility that loss-of-function mutations, promoter region DNA methylation or CNV are responsible for MAF loss in LSCC. Finally, we identified genes involved in the regulation of apoptosis harboring the MAF binding motif in their promoter region by applied FIMO and DAVID GO analysis. Our results highlight the role of miR-1290 in suppressing MAF expression in LSCC. Furthermore, MAF loss or mislocalization in FFPE LSCC tumor samples might suggest that MAF acts as a LSCC tumor suppressor by regulating apoptosis.

## 1. Introduction

Deregulation of miRNA expression is a known hallmark of human neoplasms. As with their protein-coding counterparts, miRNAs can have either tumor-suppressive or oncogenic functions and significantly contribute to cancer-associated deregulation of such processes as apoptosis, proliferation, epithelial-mesenchymal transition and cell cycle. Several miRNAs have also been demonstrated to have roles in the development of head and neck squamous cell carcinomas (HNSCC), and these include overexpressed miR-21, which is associated with poor survival rate [1]. Chang et al. found that miR-21 is involved in cell growth and proliferation activation [2]. Recurrently downregulated miRNAs, such as miR-34a and miR-34c, were also noted in HNSCC. Attenuated expression of these miRNAs in laryngeal squamous cell carcinoma (LSCC) contributes to the overexpression of *CCND1* and *c-MET* respectively, resulting in activation of cell growth and invasiveness. Thus, an analysis of miRNAs and their target genes could implicate novel players in the process of carcinogenesis. 

In our previous published study, we used this strategy to describe a new potential oncomir in LSCC, namely, miR-1290. Induced inhibition of miR-1290 resulted in increased MAF protein levels, suggesting a direct interaction between the miRNA and the *MAF* mRNA [3]. MAF is an interesting novel tumor suppressor candidate that belongs to a transcription factor family comprising seven proteins. These proteins are divided into large MAF (MAFA, or L-MAF), MAFB and MAF (also known as c-MAF), NRL (neural retina leucine zipper), and small MAF, MAFF, MAFG and MAFK. Interestingly, several reports suggest an oncogenic role for MAF [4,5,6]. For example, overexpression of *MAF* is a frequent oncogenic event in multiple myeloma, triggering pathological bone marrow stromal cell interactions and promoting proliferation [7]. However, given our previous findings, we speculate that it can also act as a tumor suppressor, depending on the cell type [8]. Therefore, in this study, we further analyze its potential involvement as a suppressor in LSSC.

We focus on verifying whether miR-1290 in fact directly interacts with the 3′UTR of *MAF*. Moreover, we analyze the distribution of the MAF protein in 128 paraffin-embedded LSCC samples and perform FIMO and Gene Ontology (GO) analysis in order to locate potential genes and biological processes deregulated by MAF loss in LSCC.

## 2. Materials and Methods

### 2.1. Cell Lines

The HEK 293T cell line (authenticated by STR DNA profiling) and the LSCC cell lines were cultured at 37 °C under 5% CO_2_ atmosphere in Dulbecco’s modified Eagle medium supplemented with 10% fetal bovine serum. LSCC cell lines (Supplementary Table S1) were obtained from the University of Turku (Turku, Finland).

### 2.2. LSCC Primary Samples

LSCC samples (n = 18) were collected during laryngectomy from patients treated at the Department of Otolaryngology, University of Medical Sciences in Poznan (Supplementary Table S2). Fresh frozen samples were used for RNA isolation. 

### 2.3. LSCC Paraffin Samples

Archived formalin-fixed and paraffin-embedded tissue sections of LSCC tumors were obtained from the Department of Clinical Pathomorphology, Collegium Medicum in Bydgoszcz of the Nicolaus Copernicus University in Torun. The study group included samples from 128 patients (111 men, 17 women) (Table 1). 

The control group consisted of disease-free normal mucosa samples, which were collected at least 2 cm from the tumor (19 cases). The results of the analysis had no effect on standard diagnostic and therapeutic procedures.

### 2.4. Dual-Luciferase Reporter Assay

The TargetScanHuman (release 7.1) database was used to identify putative miR-1290 binding sites in the *MAF* 3′UTR [9]. Four *MAF* 3′UTR fragments containing single binding sites for miR-1290 as well as SacI and XbaI ends were synthesized (Genomed, Warsaw, Poland) (Table 2). 

The oligonucleotides containing wild-type (WT) or mutated (MUT) binding sites flanked by 30 bp were designed as described by Mets et al. [10]. Oligonucleotides were cloned to the pmirGLO Dual-Luciferase miRNA Target Expression Vector (Promega, Madison, WI, USA), purified using the PhasePrep BAC DNA Kit (Sigma-Aldrich, St. Louis, MO, USA) and verified by Sanger sequencing (primer sequences are shown in Table 3).

To verify the miR-1290 *MAF* 3′UTR binding, the HEK 293T cell line was co-transfected with the following constructs using jetPRIME DNA/siRNA (Polyplus-transfection, Illkirch-Graffenstaden, France) reagents:

125 ng of vector containing the 3′UTR WT sequence + 50 nM of the analyzed miRNA mimic (mirVana^®^ miRNA mimic, MC13679, Invitrogen, Waltham, MA, USA).

125 ng of vector containing the 3′UTR WT sequence + 50 nM of the mimic negative control (NC) (mirVana™ miRNA Mimic, Negative Control #1, Invitrogen, Waltham, MA, USA).

125 ng of vector containing the 3′UTR MUT sequence + 50 nM of the analyzed miRNA mimic (mirVana^®^ miRNA mimic, MC13679, Invitrogen, Waltham, MA, USA).

125 ng of vector containing the 3′UTR MUT sequence + 50 nM of the mimic negative control (NC) (mirVana™ miRNA Mimic, Negative Control #1, Invitrogen, Waltham, MA, USA).

The firefly and *Renilla* luciferase activity were measured on a GloMax^®^ 96 Microplate Luminometer (Promega, Madison, WI, USA).

### 2.5. IHC Staining

The tissue microarray (TMA) was prepared as previously described [11]. Consecutive 3 µm thin TMA tissue sections were cut and used for immunohistochemical staining, as reported previously [12]. Tissue sections were incubated with primary rabbit polyclonal anti-MAF antibody overnight at 4 °C (cat. No: ab77071; dilution 1:400; Abcam, Cambridge, UK). Staining was standardized using a series of positive and negative control reactions, and the presence of the analyzed antigen was evaluated in human normal colon tissue. Nuclear staining in glandular cells and in T lymphocytes was considered to indicate positive *MAF* expression. Negative control reactions were performed by substituting the primary antibody with a solution of 1% BSA (Sigma-Aldrich; St. Louis, MO, USA) diluted in PBS (Agilent, DAKO; Glostrup, Denmark). MAF protein expression was evaluated according to morphometric principles based on a Remmele-Stegner scale (IRS—Index Remmele-Stegner; immunoreactive score) [13]. Morphologic examination was performed at 20× original objective magnification using the ECLIPSE E400 (Nikon Instruments Europe, Amsterdam, Netherlands) light microscope. For evaluating MAF expression, immunohistochemical reactions were scored on a two-point qualitative scale: 0, indicating a complete absence of MAF staining in all neoplastic cells or only cytoplasmic MAF in neoplastic cells, and 1, indicating nuclear staining in all neoplastic cells. 

### 2.6. Bisulfite Pyrosequencing

DNA from LSCC cell lines was obtained using phenol/chloroform extraction and ethanol precipitation. The EZ DNA Methylation-Gold™ kit (Zymo Research, Freiburg im Breisgau, Germany) was used to perform DNA bisulfite conversion of the LSCC cell lines (Supplementary Table S1). The assay for bisulfite sequencing of the *MAF* promoter region was designed using the PyroMark Assay Design Software 2.0.1.15 (Qiagen; Hilden, Germany) (Table 2). The amplified sequence of 131 bp (GRCh38/hg38 chr16:79,600,881–79,601,012) covered 3 CpG dinucleotides: CpG 1 chr16:79,600,977–79,600,978, CpG 2 chr16:79,600,974–79,600,975 and CpG 3 chr16:79,600,967–79,600,968. The PyroMark PCR kit was used to prepare the following reaction mixture: 12.5 µL PyroMark Master Mix, 2.5 µL CoralLoad, 0.5 µL of F and R primer (20 pmol/µL), 1 µL of converted DNA (25 ng/µL) and 8 µL H_2_O. The PCR conditions were as follows: 95 °C for 15 min × 1; (94 °C for 30 s, 55 °C for 30 s, 72 °C for 30 s) × 45; 72 °C for 10 min × 1; 4 °C ∞. PCR products were visualized on 2% agarose gel stained by SimplySafe (EURx; Gdansk, Poland) under UV light (BioDoc-it Imaging System, UVP, Upland, CA, USA). Pyrosequencing was performed using the PyroMark Q24 (Qiagen; Hilden, Germany) sequencer, as described previously [14]. Each run included fully methylated (M—commercially available methylated DNA, Millipore, Hilden, Germany) and unmethylated controls (UM—whole-genome amplified DNA from pooled peripheral blood lymphocytes by using the GenomePlex Complete Whole Genome Amplification (WGA) kit (Sigma-Aldrich, St. Louis, MO, USA). Mean DNA methylation level was assessed for the three analyzed CpG dinucleotides. 

### 2.7. Mutation Screening and DNA Methylation Analysis of MAF by TCGA Database Mining

Data generated by the TCGA Research Network were used to identify potential mutations of *MAF* and to further analyze the DNA methylation profile of the *MAF* promoter region as well as to verify the expression of both *MAF* isoform [15]. For the mutation screen, the analyzed cohort of samples consisted of 111 laryngeal primary tumor cases (TCGA-HNSC project). DNA methylation levels of *MAF* were obtained by downloading microarray beta values from 117 laryngeal primary tumor cases (R package TCGAbiolinks) [16]. We used Illumina methylation probes (Supplementary Table S3) associated with the gene, as shown in the UCSC Table Browser, to visualize the methylation profile of *MAF*.

### 2.8. MAF Copy Number Variation Analysis

CGH profiles from 13 LSCC cell lines from our previous study [17,18] were used to screen for potential *MAF* copy-number alterations. A mean log2 ratio value between 0.5 and −0.5 for the MAF-associated array tags (Supplementary Table S4) was regarded as a normal copy number.

### 2.9. FIMO Analysis 

The MAF consensus binding motif (vdwdnTGCTGAbdhddvhd) was downloaded from the HOCOMOCO ChIP-Seq database [19]. The promoter sequences of predicted MAF targets/genes (up to 1000 bases upstream of the gene) were download from the UCSC Table Browser (GRCh38/hg38), as described by Karolchik et al. [20]. Binding motif enrichment analysis for c-MAF predicted targets was performed using FIMO from the MEME package [21]. FIMO analysis was performed with the following parameters: # Scan: DNA motif on both strands; # Match *p*-value < 1E^−4^. Only sequences with at least one consensus binding motif with a *p*-value < 1E^−4^ were considered as possible MAF targets. The RefSeq IDs obtained from the analysis were converted into gene symbols using Biotools [22].

The genes retrieved by FIMO were used for GO analysis using the DAVID (6.7) tool [23] to obtain the set of biological processes potentially involved in LSCC pathogenesis. In addition, a set of all downregulated genes in 5 LSCC cell lines (UT-SCC-107, UT-SCC-116, UT-SCC-22, UT-SCC-34, UT-SCC-4) with the lowest expression of *MAF* (expression microarray results with detection signal *p*-value < 0.05, as described previously [17]) was used as a background. This contrasts with the typical set used in such analyses, which would include all known human genes. The approach used in this study improves the accuracy of the analysis and reduces the number of potential false results.

### 2.10. Vector Preparation and LSCC Cell Line Transduction

The genomic sequence of pre-miRNA-1290 hairpin flanked by 100–250 nt on each site was amplified using specific primers (Table 2), designed as described in Kluvier at al. and Paczkowska et al. [24,25]. The PCR product with sticky ends was cloned into the pCDH-CMV-MCS-EF1α-GreenPuro vector (SBI, Palo Alto, CA, USA). The lentiviral particles containing the empty vector or vector with pre-miRNA-1290 were harvested 48 h after transfection of HEK 293T cells. Two LSCC cell lines, UT-SCC-34 and UT-SCC-11, were transduced by the vector carrying the miR-1290 sequence as well as by the empty vector. Cells with stable expression of miRNA-1290 were selected by puromycin. After 7 days of antibiotic selection, transduction efficiency was analyzed by measurement of GFP expression using flow cytometry (CytoFLEX, Beckman Coulter, Indianapolis, IN, USA). Cultures containing >80% of transduced cells were used for RNA isolation.

### 2.11. Real-Time qPCR

Total RNA from transduced cell lines was isolated with the use of Trizol reagent based on the method developed by Chomczynski, described elsewhere [26]. cDNA for miR-1290 expression analyses was synthesized with the universal cDNA synthesis kit according to the supplier’s protocol (Exiqon, Vedbaek, Denmark). LNA-modified primers (Qiagen, Vedbaek, Denmark) for detection of miR-1290 and referenced U6 snRNA were used for real-time qPCR (Table 3). The sequences of primers were not provided by the manufacturer. Each reaction was performed in triplicate on the CFX qPCR Instrument (BioRad, Hercules, CA, USA) with the use of SybrGreen Mastermix (Exiqon, Vedbaek, Denmark).

cDNA synthesis was performed using 2 µg of total RNA, which was reverse-transcribed by the Maxima First-Strand cDNA Synthesis Kit for RT-qPCR (Thermo Scientific, Waltham, MA, USA) with dsDNase, according to manufacturer’s instructions. Primer pairs (Table 3) for the *PRODH*, *MAF NM_005360* and *MAF NM_001031804* genes as well as the reference genes (*β-ACTIN* and *GAPDH*) were designed using the PrimerBlast [27] software, as described in [28]. Each reaction was performed in triplicate on the CFX qPCR Instrument (BioRad, Hercules, CA, USA) with the use of HOT FIREPol^®^ EvaGreen^®^ qPCR Mix Plus (no ROX) (Solis BioDyne, Tartu, Estonia). Amplification was conducted in a total volume of 10 µL containing 2 µL of EvaGreen, 0.4 µM of each primer and 1 µL of mRNA (50 ng/µL) in the following conditions: 95 °C for 900 s; 40 cycles of: 95 °C for 20 s, 55 °C for all analyzed genes for 10 s, 72 °C for 20 s; 1 × 95 °C for 30 s; 1 × 50 °C for 30 s. The specificity of the product was verified by generating the melting curve by heating the samples from 50 to 95 °C in 0.5 °C increments, with a dwell time at each temperature of 10 s (0.5 °C for 10 s). The BioRad Genex application v1.10 was used to calculate the relative expression of selected genes (in relation to the references genes) based on automatically generated background values for threshold cycle determination (Ct).

### 2.12. Statistics

IHC staining results were analyzed using the chi-square test of independence with Yates’s correction using Stats package [29]. Luciferase assay results were analyzed using a two-tailed t-test. *p*-values below 0.05 were considered statistically significant.

## 3. Results

### 3.1. MiR-1290 Interacts with the 3′UTR of MAF

**Figure 1:**
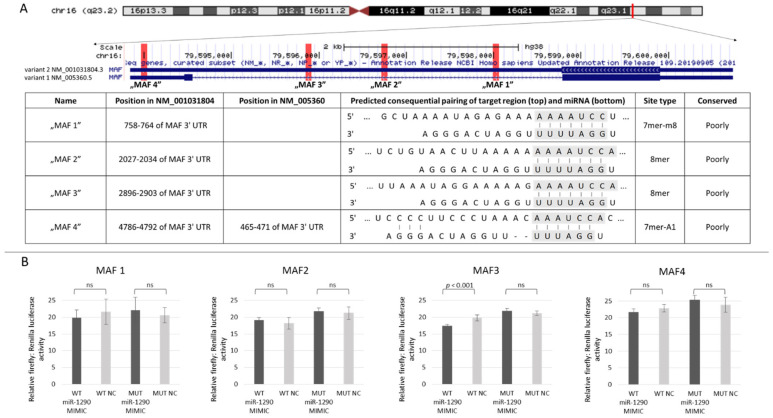
Characterization of has-miR-1290 binding sites in the 3′UTR of MAF (**A**). Luciferase reporter assay results for four hsa-miR-1290 binding sites in the *MAF* 3′UTR (ns = not significant) (**B**).

In our previous study, we demonstrated that inhibition of miR-1290 results in increased MAF protein levels in LSCC cell lines [3]. In order to confirm the presence of a direct interaction, we first used the Target Scan tool to delineate binding sites of miR-1290 in the 3′UTR sequence of *MAF*. Of the four binding sites identified, one is common to both transcript variants 1 (NM_005360.5) and 2 (NM_001031804.3). The remaining three binding sites, meanwhile, were localized exclusively in transcript variant 2 (Figure 1A). We then evaluated the expression level of both isoforms in LSCC cell lines and tumors (Supplementary Figure S1) and confirmed that *MAF* NM_005360.5 as well as *MAF* NM_001031804.3 are expressed in these samples. 

Using the dual reporter assay, we demonstrated that miR-1290 represses only one of the four identified binding sites, namely, “MAF3” (Figure 1B). For this interaction, the firefly luciferase signal in cells transfected by the WT MAF3–miR-1290 mimic compared to WT MAF3–NC decreased by 12.28%, a significant difference (*p* < 0.0001), while the change for the MUT MAF3–miR-1290 mimic compared to MUT MAF3–NC was not significant (*p* > 0.05). This binding site (MAF3) is localized in the transcript variant 2 (NM_001031804.3). The firefly luciferase signal changes for the other three binding sites were not significant (Figure 1B). Thus, we have demonstrated that miR-1290 directly regulates *MAF* (NM_001031804.3) expression by interacting with the *MAF* 3′UTR through the binding site located at chr16:79,595,878–79,595,885 (GRCh38/hg38).

### 3.2. LSCC Samples Are Characterized by the Absence of Nuclear Expression of MAF

**Figure 2:**
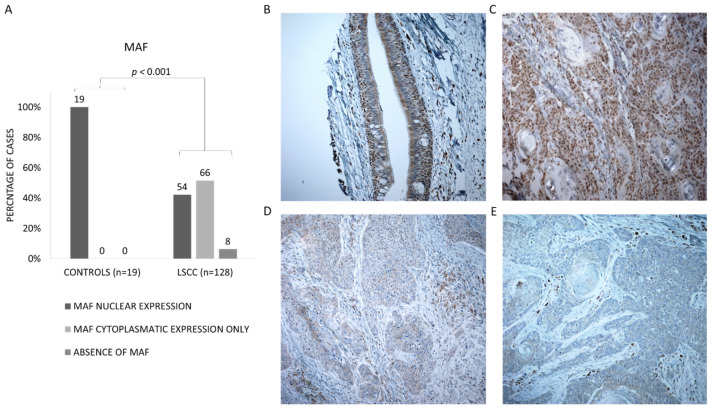
Immunohistochemical staining of MAF in LSCC. (**A**) Cellular distribution of MAF protein in normal mucosa and primary LSCC. (**B–E**) Representative microphotographs of MAF immunohistochemical staining (brown color = positive reaction). (**B**) Nuclear MAF expression in normal mucosa, (**C**) LSCC tumors with nuclear c-MAF expression, (**D**) LSCC tumors with cytoplasmic MAF expression, (**E**) LSCC tumor with absence of MAF expression.

To search for potential MAF loss and to analyze its cellular distribution in LSCC, we evaluated MAF protein expression in 128 formalin-fixed, paraffin-embedded tumor samples from LSCC patients, as well as expression in the control group that consisted of 19 normal mucosa cases. Based on the protein atlas data [30], as well as our results from the control setups, we assumed that normal MAF expression is observed in the nucleus with co-expression in the cytoplasm. In normal, non-tumor mucosa, MAF showed normal nuclear and cytoplasmatic expression in all analyzed cases (19/19; 100%) (Figure 2B). However, in LSCC, 74/128 (58%) cases demonstrated aberrant expression of MAF, where in 8/74, MAF was entirely absent in cancer cells (Figure 2E), and in 66/74, only cytoplasmatic expression was observed (Figure 2D). The remaining 54/128 (42%) cases showed normal nuclear and cytoplasmatic expression of the protein (Figure 2B). Importantly, differences in MAF expression in these two groups (tumors vs. controls) are statistically significant (*p* < 0.001) (chi square with Yates’ correction) (Figure 2A).

### 3.3. Decreased Expression of MAF in LSCC Is Not Associated with Hypermethylation of Promoter nor Mutations nor with Changes in MAF Gene Copy Number

To verify if any mechanism other than epigenetic repression by miRNA was responsible for the decreased expression of MAF in LSCC, a combined analysis of *MAF* mutation screening, promoter region DNA methylation and CNV was performed. We screened for *MAF* mutations using the TCGA data, but surprisingly, in the 111 laryngeal cancer cases, no mutation in the *MAF* gene was found. Moreover, there were only two samples with *MAF* alterations (chr16:g.79598780C>A; chr16:g.79599782G>A; GRCh38/hg38) in the remaining 396 HNSCC samples from different primary tumor sites. Additionally, in our own analyses, we found no elevated methylation of the CpG dinucleotides within the *MAF* promoter region in the 21 LSCC cell lines through bisulfite pyrosequencing (mean methylation equal to 4.88, SD = 1.5). Similarly, TCGA methylation data from 117 LSCC cases for 19 tags localized within a CpG island near the *MAF* promoter region (GRCh37/hg19 chr16:79632316–79635445) showed a lack of DNA methylation (mean methylation equal to 6.35, SD = 2.63). This finding confirmed our observation that methylation is not the mechanism responsible for *MAF* downregulation in LSCC. Similarly, we found no copy-number alterations of *MAF* in the CGH profiles of 16 LSCC cell lines from our previous study [17]. Therefore, the miRNA-mRNA crosstalk is the only mechanism of MAF downregulation in LSCC observed in our analyses.

### 3.4. Potential Impact of MAF on Regulation of Apoptosis by Binding to Promoter Regions of Apoptosis-Related Genes

**Figure 3:**
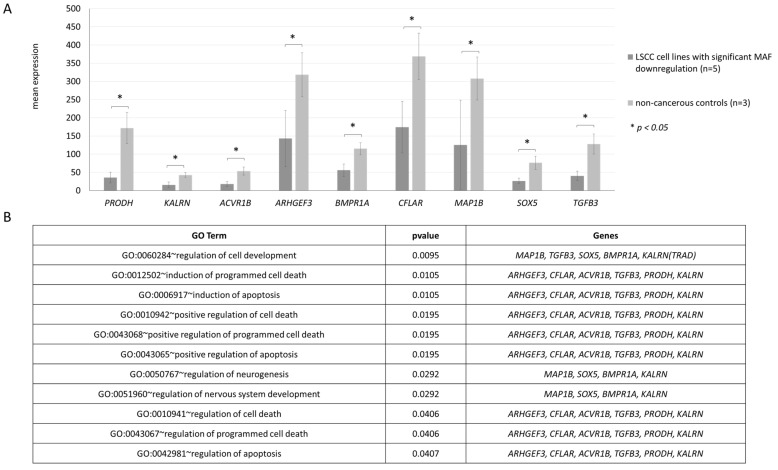
Results of combined FIMO and DAVID GO analysis. (**A**) Downregulated genes in LSCC (expression microarray data), with MAF binding motif in the promoter sequence and a putative role in apoptosis regulation. Human larynx total RNA (Stratagene, Agilent Technologies, Waldbronn, Germany) and total RNA from the bronchial airway epithelia reconstituted in vitro (Epithelix Sarl, Geneve, Switzerland) as well as normal mucosa from surgical margin were used as non-cancerous controls. (**B**) Processes regulated by selected genes, related to apoptosis and cell development, potentially downregulated as a consequence of MAF loss in LSCC.

The ubiquitously expressed MAF is involved in the transcriptional activation of various genes. Thus, we aimed to identify MAF-regulated genes with potential roles in LSCC pathogenesis. As *MAF* is downregulated in LSCC, we searched for genes downregulated in LSCC with MAF binding motifs in their promoter regions. For this purpose, we selected the 5/16 LSCC cell lines (based on microarray expression data described previously [17]) with the lowest *MAF* expression and conducted FIMO analysis on the set of 672 genes downregulated in these 5 LSCC cell lines. FIMO analysis revealed 451 genes with MAF binding motif, of which 63 had at least one motif in the promoter region (Supplementary Table S5). To verify if these 63 genes, potentially regulated by MAF, are engaged in the pathogenesis of LSCC, we conducted the GO analysis, which revealed 11 processes with 9 assigned genes related to cell development and apoptosis (Figure 3). Even though most of these genes are described as antiapoptotic factors in other cancers [31,32,33,34], we cannot exclude that their activity may differ depending on the cell context and tissue specificity. Therefore, from this group of genes, we have chosen *PRODH,* a TP53-related proapoptotic agent [35], as the best candidate for further functional verification.

**Figure 4:**
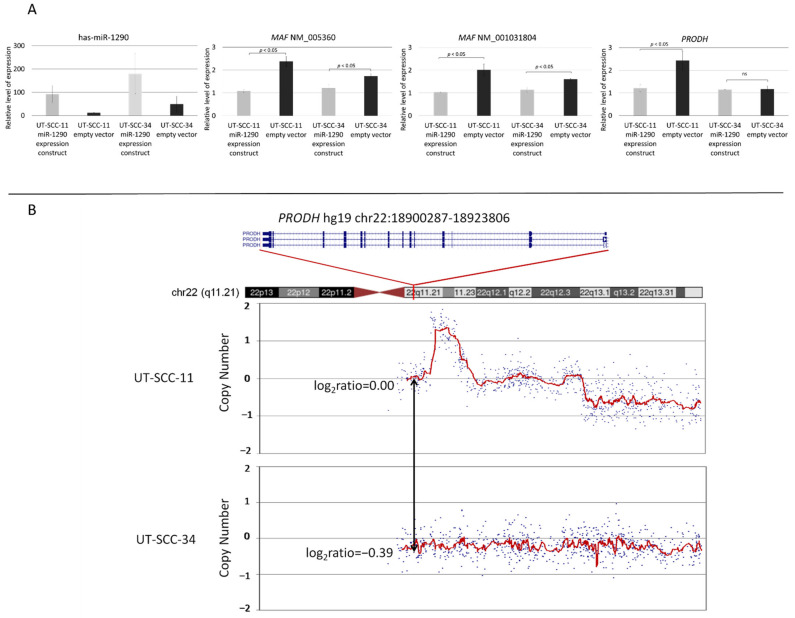
Expression level of *MAF* NM_005360, *MAF* NM_001031804 and *PRODH* genes in LSCC cell lines after transduction by miR-1290 expression vector or empty vector (**A**). Copy-number plots based on array CGH data for chromosome 22 in UT-SCC-11 and UT-SCC-34 cell lines [17,18]. The red line on the plots represents the mean copy number. The black arrow points to *PRODH*-associated array tag (**B**). ns: not significant.

In order to verify the *in silico* analysis, we overexpressed miR-1290 by transducing two LSCC cell lines (UT-SCC-11 and UT-SCC-34) with the respective expression construct. We hypothesized that additional miR-1290 transcripts will strengthen the downregulation of *MAF,* which in turn will result in decreased expression of *PRODH*, a MAF-regulated gene. We have established two cell lines with stable overexpression of miR-1290 (Figure 4A) and observed significant downregulation of both isoforms of *MAF.* Fold change (cells transduced by miR-1290 expression construct versus empty vector) of *MAF* NM_005360 was 0.45 for UT-SCC-11 and 0.69 for UT-SCC-34, while of *MAF* NM_001031804 was 0.71 and 0.52, respectively. We further tested whether, along with our hypothesis, decreased expression of *MAF* has an influence on *PRODH* transcription. Indeed, we demonstrated that in UT-SCC-11, *PRODH* expression was reduced by approximately 50% (Figure 4A). However, regardless of the significant reduction of *MAF* expression in UT-SCC-34, we did not observe any subsequent differences in *PRODH* expression. To further elucidate this finding, we analyzed our previous array CGH results [17,18], for potential copy-number alterations of the *PRODH* gene in the UT-SCC-11 and UT-SCC-34 cell lines (Figure 4B). We observed loss of *PRODH* DNA (log2ratio = −0.39) in UT-SCC-34. These array CGH data indicate that chromosomal alterations within chromosome 22 might result in downregulation of *PRODH,* independently from MAF regulation.

These results suggest that the loss of MAF in LSCC may contribute to deregulation of apoptosis in the neoplastic cells via changes of *PRODH* expression.

## 4. Discussion

In our previous study, we demonstrated that *MAF* is downregulated at the mRNA level in LSCC cell lines and tumor samples, and we identified the putatively oncogenic miR-1290 to be a regulator of the level of MAF protein in the analyzed LSCC cell lines. Here, we shed further light on this finding by demonstrating the direct interaction between miR-1290 and *MAF* NM_001031804. Surprisingly, after miR-1290 overexpression, we observed a significant reduction in expression of both *MAF* isoforms. That fact could be explained in several ways. First of all, downregulation of *MAF* NM_001031804 as well as miR-1290 overexpression may cause altered expression of other *MAF* regulators, which influence both *MAF* isoforms. Alternatively, there might be other bindings sites for miR-1290 in the 3′UTR of *MAF* NM_005360 than those found in the TargetScan database. Moreover, we show a recurrent loss of MAF protein in formalin-fixed, paraffin-embedded LSCC sections. In an attempt to explain this observation, we excluded such cancer-related mechanisms as loss-of-function mutations or promoter DNA hypermethylation as being responsible for the downregulation of *MAF* in LSCC. We are aware that other mechanisms such as deregulation of transcription factors or changes in *MAF* promoter sequence can be implicated in MAF downregulation. Furthermore, other miRNAs could also regulate MAF. One of such candidates was indicated in our previous study [3], where we found that the well-known oncomir miR-21-3p has a binding site in the 3′UTR of *MAF* (data not shown). Nevertheless, the described findings highlight the importance of the experimentally validated role of miR-1290 in silencing *MAF* in LSCC. In addition, we observed mislocalization of MAF protein in LSCC compared to controls. This finding signifies the complexity of MAF status in LSCC as changes of subcellular localization of proteins are described as a typical cancer-related phenomenon [36]. Elucidation of the exact mechanism responsible for MAF mislocalization in LSCC requires further studies focused on protein modification, signaling pathways and other related processes. 

Additionally, using *in silico* analysis, we attempted to reveal the potential role of MAF in LSCC pathogenesis. *MAF* encodes a transcription factor with a well-described oncogenic function in hematological malignancies [37], and has been shown to be a recurrent target for translocations and/or overexpression with potential consequences on the cell-cycle, proliferation and multiple myeloma growth [38]. However, MAF has also been shown to have cell-context-dependent functions, as demonstrated recently by Pouponnot et al. [8]. In line with these findings, our data support the notion that MAF may play a dual role in human neoplasms and function either as an oncogene or as a suppressor. One potential suppressive role of MAF, for instance, was described in prostate cancer [39]. Moreover, there is evidence that mouse *tp53* harbors an evolutionarily conserved binding site for MAF in the promoter region [40]. MAF-mediated activation of TP53 might therefore result in increased apoptosis, thus acting in a tumor-suppressive manner. 

This hypothesis is supported by the results of the *in silico* analyses (FIMO combined with DAVID GO) performed in our study. Using this approach, we identified 62 genes with at least one MAF binding motif in the promoter sequence. The subsequent GO analysis showed that within this group, nine genes (*PRODH, KALRN, ACVR1B, SOX5, TGFB3, CFLAR, BMPR1A, MAP1* and *ARHGEF3*) could be involved in regulation of apoptosis and cell development. Among the nine genes identified in the GO analysis, there were the *ARHGFE3* and *CFLAR*, which are inhibitors of apoptosis and show a clear oncogenic function [32,33]. However, MAF may also potentially induce the expression of such genes as *PRODH*, that trigger apoptosis [35], which was indirectly shown in the performed miR-1290 overexpression experiments in the UT-SCC-11 cell line, where we observed a reduction in *MAF* as well as *PRODH* expression level.

Finally, we demonstrated the loss of MAF protein in formalin-fixed, paraffin-embedded LSCC tumors. As much as 58% of analyzed LSCC cases showed either complete absence of MAF or lack of nuclear MAF expression, while 100% of non-tumor tissue revealed nuclear MAF expression through immunohistochemistry. This finding is in line with the assumption that in a normal tissue, a functional transcription factor should be observed in the nucleus.

## 5. Conclusions

In summary, we demonstrated that the overexpression of the miR-1290 is to a large extent responsible for MAF loss in LSCC. Moreover, in most LSCC cases, we observed another phenomenon of MAF deregulation, namely, accumulation of MAF in cytoplasm in the absence of nuclear expression. This finding emphasizes that MAF activity as a transcription factor is disrupted in LSCC. Based on these results, we also suggest that MAF may show a suppressive role in this tumor via regulation of apoptosis. Together, these findings may contribute to a better understanding of LSCC pathogenesis.

## Figures and Tables

**Table 1:** Characterization of LSCC (laryngeal squamous cell carcinoma) patients. Tumor size and lymph node status according to pathological (p) TNM (T-tumor; N-lymph node involvement; M-distant metastases) classification (7th edition) by UICC (The Union for International Cancer Control).

Parameters		Number	Percent (%)
Gender	Male	111	86.7%
	Female	17	13.3%
Age	>60	61	47.7%
	<60	67	52.3%
Tumor grade	G1	10	7.8%
	G2	107	83.6%
	G3	10	7.8%
T classification	pT1	1	0.8%
	pT2	0	0.0%
	pT3	88	68.8%
	pT4	39	30.5%
Lymph node status	pN0	71	55.5%
	pN ≠ 0	57	44.5%

**Table 2:** Sequences of oligonucleotides cloned to the pmirGLO Dual-Luciferase miRNA Target Expression Vector. The consensus motif recognized by the miRNA seed region is shown in capital letters.

Oligo	Forward	Reverse
MAF 1 WT	5′ cttttagcattgttatgctaaaatagagaaa- -AAAATCC- -tcatgaaccttccacaatcaagcctgcatct 3′	5′ ctagagatgcaggcttgattgtggaaggttcatga- -GGATTTT- -tttctctattttagcataacaatgctaaaagagct 3′
MAF 1 MUT	5′ cttttagcattgttatgctaaaatagagaac- -ACCCTCC- -tcatgaaccttccacaatcaagcctgcatct 3′	5′ ctagagatgcaggcttgattgtggaaggttcatga- -GGAGGGT- -gttctctattttagcataacaatgctaaaagagct 3′
MAF 2 WT	5′ ctgcagaactggattttctgtaacttaaaaa- -AAAATCCA- -cagttttaaaggcaataatcagtaaatgttt 3′	5′ ctagaaacatttactgattattgcctttaaaactg- -TGGATTTT- -tttttaagttacagaaaatccagttctgcagagct 3′
MAF 2 MUT	5′ ctgcagaactggattttctgtaacttaaaac- -ACCCTCCA- -cagttttaaaggcaataatcagtaaatgttt 3′	5′ ctagaaacatttactgattattgcctttaaaactg- -TGGAGGGT- -gttttaagttacagaaaatccagttctgcagagct 3′
MAF 3 WT	5′ ctgaagatcatttgtcttaaataggaaaaag- -AAAATCCA- -ctccttacttccatatttccaagtacatatt 3′	5′ ctagaatatgtacttggaaatatggaagtaaggag- -TGGATTTT- -ctttttcctatttaagacaaatgatcttcagagct 3′
MAF 3 MUT	5′ ctgaagatcatttgtcttaaataggaaaaag- -CACCGCCA- -ctccttacttccatatttccaagtacatatt 3′	5′ ctagaatatgtacttggaaatatggaagtaaggag- -TGGCGGTG- -ctttttcctatttaagacaaatgatcttcagagct 3′
MAF 4 WT	5 ′caaatagatattcgactccccttccctaaac- -AAATCCA- -cgggcagaggctccagcggagccgagcccct 3′	5 ′ctagaggggctcggctccgctggagcctctgcccg- -TGGATTT- -gtttagggaaggggagtcgaatatctatttgagct 3′
MAF 4 MUT	5 ′caaatagatattcgactccccttccctaaac- -CACGACA- -cgggcagaggctccagcggagccgagcccct 3′	5 ′ctagaggggctcggctccgctggagcctctgcccg- -TGTCGTG- -gtttagggaaggggagtcgaatatctatttgagct 3′

**Table 3:** Primer sequences used in the study.

Technics	Primer	Sequence
Bisulfite pyrosequencing	Forward (biotinylated)	5′ Biotin-GTTGTTAATTAGGGTTTAATTAGTTGAT 3′
	Reverse	5′ AAAAAAACTCCTTCCCCTCTTACA 3′
	Sequencing	5′ CCCTCTTACACCAAACTTTAC 3′
Sanger sequencing	pmirGLO_F	5′ AACACCCCAACATCTTCGAC 3′
	pmirGLO_R	5′ CTTTCGGGCTTTGTTAGCAG 3′
Real-Time qPCR	*MAF* NM_005360 Forward	5′ AATACGAGAAGTTGGTGA 3′
	*MAF* NM_005360 Reverse	5′ TTTGTGAACACACTGGTA 3′
	*MAF* NM_001031804 Forward	5′ AATACGAGAAGTTGGTGAG 3′
	*MAF* NM_001031804 Reverse	5′ ACTTATCAGGGTGGCTAG 3′
	*PRODH* Forward	5′ ACGAATAAGCGGGACAAGCA 3′
	*PRODH* Reverse	5′ CCGTCATCGCTGACTCTACC 3′
	*β*-*ACTIN* Forward	5′ CACCACACCTTCTACAATG 3′
	*β*-*ACTIN* Reverse	5′ TAGCACAGCCTGGATAG 3′
	*GAPDH* Forward	5′ GTCGGAGTCAACGGATT 3′
	*GAPDH* Reverse	5′ CCTGGAAGATGGTGATGG 3′
	has-miR-1290 miRCURY LNA miRNA PCR Assay	GeneGlobe ID—P02118634; Catalog No.—339306
	U6 snRNA miRCURY LNA miRNA PCR Assay	GeneGlobe ID—YP00203907; Catalog No.—339306

## Data Availability

Data supporting the reported results can be found at https://portal.gdc.cancer.gov/projects/TCGA-HNSC.

## Supplementary Materials

The following are available online at https://www.mdpi.com/article/10.3390/biom11071035/s1, Figure S1: Expression level of both *MAF* isoforms in LSCC cell lines and tumors analyzed by real-time qPCR (A) as well as in 100 LSCC tumors from TCGA database (B). RPKM: Reads Per Kilobase Million. Table S1: Characterization of LSCC cell lines used for bisulfite pyrosequencing, Table S2: Characterization of LSCC primary samples used for real-time qPCR, Table S3: Illumina methylation probes with annotated position and mean methylation level in 111 laryngeal primary tumor cases (TCGA-HNSC project), Table S4: *MAF*-associated CGH array tags, Table S5: List of genes with at least one MAF binding motif in the promoter region.
