# Supplementary material for: Loss of the MAF Transcription Factor in Laryngeal Squamous Cell Carcinoma
Source: Biomolecules. 2021 Jul 15;11(7):1035. doi: 10.3390/biom11071035 (PMC8301809; doi:10.3390/biom11071035)
Supplement: Supplementary file 1 [file biomolecules-11-01035-s001.zip › biomolecules-1138689-supplementary.pdf]

Supplementary Materials

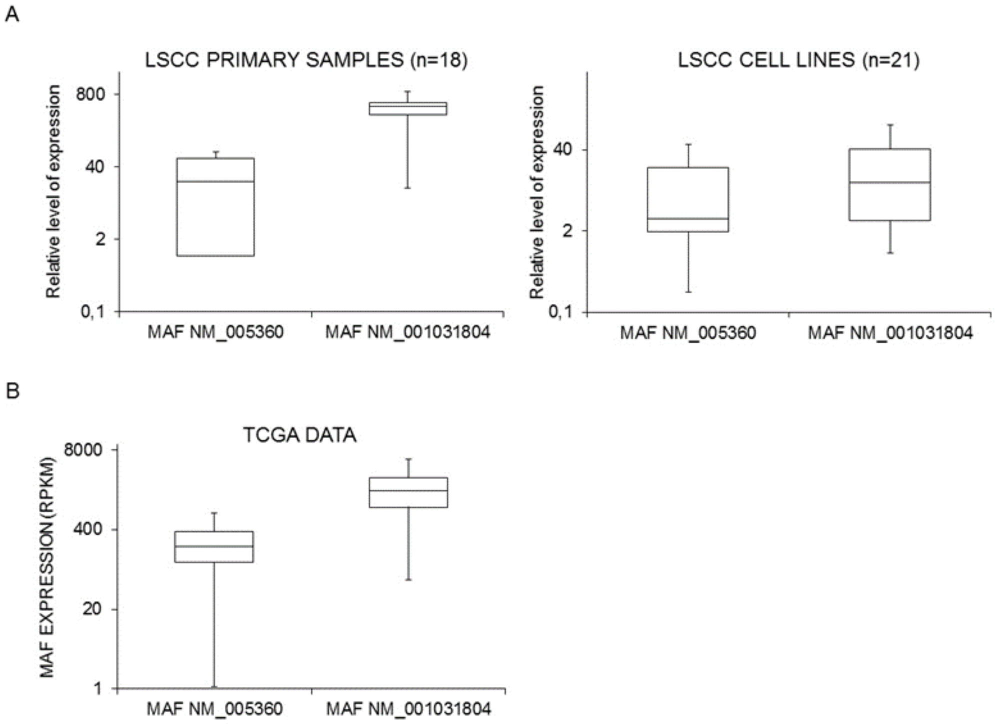

Figure S1. Expression level of both *MAF* isoforms in LSCC cell lines and tumors analyzed by real-time qPCR (A) as well as in 100 LSCC tumors from TCGA database (B). RPKM: Reads Per Kilobase Million.

Table S1. Characterization of LSCC cell lines used for bisulfite pyrosequencing. UT-SCC: University of Turku – Squamous Cell Carcinoma; M: male; F: female; TNM: TNM classification (T-tumor; N-lymph node involvement; M-distant metastases); Pri - primary tumor; Rec - recurrence; Met - metastasis; Per - persistent tumor; G - tumor grade, NA - data not available.

Cell line	Sex	Age (yrs)	Primary tumorlocation	TNM	Specimen site	Type of lesion	Grade
UT-SCC-106A	M	59	SCC plicae vocalis	$T_{1A}N_0M_0$	larynx	pri	G2
UT-SCC-106B	M	59	SCC plicae vocalis	$rT_3N_0M_0$	larynx	rec	G3
UT-SCC-107	M	46	SCC laryngis supraglottidis	$T_4N_{2C}M_0$	larynx	pri	G2
UT-SCC-108	M	68	SCC laryngis supraglottidis	$T_2N_0M_0$	larynx	pri	G3
UT-SCC-11	M	58	glottic larynx	$T_1N_0M_0$	larynx	rec	G2
UT-SCC-113	M	50	SCC laryngis transglottica	$T_3N_0M_0$	larynx	pri	G3
UT-SCC-116	M	60	SCC laryngis supraglottidis	$T_4N_1M_0$	larynx	pri	G2
UT-SCC-13	M	53	supraglottic larynx	$T_3N_0M_0$	larynx	rec	G2
UT-SCC-19A	M	44	glottic larynx	$T_4N_0M_0$	larynx	pri	G2
UT-SCC-19B	M	44	glottic larynx	$T_4N_0M_0$	larynx	pri (per)	G2
UT-SCC-22	M	79	glottic larynx	$T_1N_0M_0$	larynx	rec	G2
UT-SCC-23	M	66	SCC transglottica	$T_3N_0M_0$	larynx	pri (per)	G1
UT-SCC-29	M	82	glottic larynx	$T_2N_0M_0$	larynx	pri	G1
UT-SCC-34	M	63	supraglottic larynx	$T_4N_0M_0$	supraglottic larynx	pri	G1
UT-SCC-35	M	50	glottic larynx	$T_2N_0M_0$	larynx	resid	G2
UT-SCC-38	M	66	glottic larynx	$T_2N_0M_0$	larynx	pri	G2
UT-SCC-50	M	70	glottic larynx	$T_2N_0;rT_2N_0$	larynx	rec	G3
UT-SCC-57	M	76	glottic larynx	$T_2N_0M_0$	larynx	rec	G1-G2
UT-SCC-6A	F	51	supraglottic larynx	$T_2N_1M_0$	larynx	rec	G1
UT-SCC-6B	F	51	supraglottic larynx	$T_2N_1M_0$	neck	met	G1
UT-SCC-75	M	56	SCC laryngis	$T_2N_{2B}M_0$	larynx	pri	G2

Table S2. Characterization of LSCC primary samples used for real-time qPCR. TNM: TNM classification (T-tumor; N-lymph node involvement; M-distant metastases); G - tumor grade, NA - data not available.

	Probe	Localization	TNM	Grade
**LSCC Primary samples**	1	Larynx	$T_4N_2M_0$	NA
	2	Larynx	$T_3N_0M_0$	G2
	3	Larynx	$T_4N_1M_0$	G1
	4	Larynx	$T_3N_1M_0$	G2
	5	Larynx	$T_4N_1M_1$	NA
	6	Larynx	$T_4N_0M_0$	G1
	7	Larynx	$T_4N_1M_0$	G2
	8	Larynx	$T_3N_1M_0$	G2
	9	Larynx	$T_4N_0M_0$	G1
	10	Larynx	$T_4N_2M_0$	G2
	11	Larynx	$T_4N_1M_0$	G1
	12	Larynx	$T_{4a}N_{2b}M_0$	G2
	13	Larynx	$T_4N_1M_0$	G2
	14	Larynx	$T_4N_3M_0$	G1
	15	Larynx	$T_4N_2M_0$	G2
	16	Larynx	$T_4N_1M_0$	G2
	17	Larynx	$T_3N_1M_0$	G2
	18	Larynx	$T_4N_1M_0$	G2

Table S3. Illumina methylation probes with annotated position and mean methylation level in 111 laryngeal primary tumor cases (TCGA-HNSC project).

Tag Name	Position (GRCh37/hg19)	Mean Methylation Level
cg00509921	chr16:79,632,332-79,632,332	0,07
cg04132983	chr16:79,632,621-79,632,621	0,15
cg06834240	chr16:79,632,626-79,632,626	0,11
cg04626565	chr16:79,632,784-79,632,784	0,08
cg07870982	chr16:79,633,041-79,633,041	0,08
cg04858597	chr16:79,633,453-79,633,453	0,05
cg07504616	chr16:79,633,543-79,633,543	0,09
cg08553999	chr16:79,633,724-79,633,724	0,08
cg06018531	chr16:79,633,902-79,633,902	0,07
cg14055374	chr16:79,634,410-79,634,410	0,03
cg06327596	chr16:79,634,583-79,634,583	0,04
cg07242563	chr16:79,634,675-79,634,675	0,02
cg06881580	chr16:79,634,709-79,634,709	0,06

cg02962380	chr16:79,634,724-79,634,724	0,05
cg10689889	chr16:79,634,732-79,634,732	0,05
cg07026599	chr16:79,634,737-79,634,737	0,03
cg09822018	chr16:79,635,284-79,635,284	0,08
cg08475576	chr16:79,635,404-79,635,404	0,05
cg01001468	chr16:79,635,425-79,635,425	0,03

Table S4. MAF associated CGH array tags.

CGH Platform	Probe Name	Position (NCBI36/hg18)
Agilent Human Genome CGH 44K	A_14_P101022	chr16:78,185,404-78,185,463
	A_14_P118163	chr16:78,185,744-78,185,803
	A_14_P118163	chr16:78,185,923-78,185,969
	A_14_P102896	chr16:78,187,931-78,187,990
	A_14_P139279	chr16:78,219,082-78,219,141
Agilent Human Genome CGH 244A	A_14_P139279	chr16:78,219,082-78,219,141
	A_16_P20533099	chr16:78,178,440-78,178,499
	A_14_P101022	chr16:78,185,404-78,185,463
	A_14_P118163	chr16:78,185,744-78,185,803
	A_14_P102782	chr16:78,185,923-78,185,969
	A_14_P102896	chr16:78,187,931-78,187,990
	A_16_P20533159	chr16:78,195,459-78,195,518

Table S5. List of genes with at least one motif in the promoter region.

Gene	Position	Chromosomal localization	Name	Locus type
ABHD14B	chr3:52002526-52008646	3p21.2	abhydrolase domain containing 14B	protein coding
ACKR4	chr3:132318981-132321485	3q22.1	atypical chemokine receptor 4	protein coding
ACVR1B	chr12:52347164-52390863	12q13.13	activin A receptor type 1B	protein coding
ACY1	chr3:52017551-52023213	3p21.2	aminoacylase 1	protein coding
ARHGEF3	chr3:56761448-56950464	3p14.3	Rho guanine nucleotide exchange factor 3	protein coding
ARPC4	chr3:9834232-9848789	3p25.3	actin related protein 2/3 complex subunit 4	protein coding
BMPR1A	chr10:88516376-88687726	10q23.2	bone morphogenetic protein receptor type 1A	protein coding
CAB39L	chr13:49882786-49987887	13q14.2	calcium binding protein 39 like	protein coding
CCDC80	chr3:112315644-112359990	3q13.2	coiled-coil domain containing 80	protein coding
CDKL5	chrX:18460344-18671749	Xp22.13	cyclin dependent kinase like 5	protein coding
CEP68	chr2:65283550-65314138	2p14	centrosomal protein 68	protein coding
CFLAR	chr2:201980887-202041410	2q33.1	CASP8 and FADD like apoptosis regulator	protein coding
CPEB4	chr5:173315352-173388983	5q35.2	cytoplasmic polyadenylation element binding protein 4	protein coding
DERL3	chr22:24176690-24181193	22q11.23	derlin 3	protein coding
DHRS9	chr2:169923587-169952677	2q31.1	dehydrogenase/reductase 9	protein coding
DMD	chrX:31137339-33357505	Xp21.2-p21.1	dystrophin	protein coding
DPP4	chr2:162848755-162930725	2q24.2	dipeptidyl peptidase 4	protein coding
EHF	chr11:34654113-34684834	11p13	ETS homologous factor	protein coding
EPC1	chr10:32556644-32667697	10p11.22	enhancer of polycomb homolog 1	protein coding
ETFBKMT	chr12:31812137-31826046	12p11.21	electron transfer flavoprotein subunit beta lysine methyltransferase	protein coding

EVL	chr14:100437759-100610573	14q32.2	Enah/Vasp-like	protein coding
FKBP1A-SDCBP2	chr20:1290553-1373816	20p13	FKBP1A-SDCBP2 readthrough (NMD candidate)	lncRNA
FUCA1	chr1:24171571-24194859	1p36.11	alpha-L-fucosidase 1	protein coding
GMPPB	chr3:49757349-49761384	3p21.31	GDP-mannose pyrophosphorylase B	protein coding
HAPLN3	chr15:89420525-89438775	15q26.1	hyaluronan and proteoglycan link protein 3	protein coding
HLA-DMB	chr6:32902413-32908805	6p21.32	major histocompatibility complex, class II, DM beta	protein coding
INAFM1	chr19:47777680-47778980	19q13.32	InaF motif containing 1	protein coding
IPCEF1	chr6:154475618-154651215	6q25.2	interaction protein for cytohesin exchange factors 1	protein coding
IRF5	chr7:128577086-128590092	7q32.1	interferon regulatory factor 5	protein coding
KALRN	chr3:123813528-124394274	3q21.2	kalirin RhoGEF kinase	protein coding
KIAA1217	chr10:24498067-24836772	10p12.2-p12.1	sickle tail protein homolog	protein coding
MAP1B	chr5:71403302-71505390	5q13.2	microtubule associated protein 1B	protein coding
MAU2	chr19:19431638-19469563	19p13.11	MAU2 sister chromatid cohesion factor	protein coding
MGAT1	chr5:180211780-180229806	5q35.3	alpha-1,3-mannosyl-glycoprotein 2-beta-N-acetylglucosaminyltransferase	protein coding
MIR4271	chr3:49311553-49311619	3p21.31	microRNA 4271	microRNA
MSRA	chr8:9911802-10286401	8p23.1	methionine sulfoxide reductase A	protein coding
MXRA7	chr17:74668633-74707047	17q25.1	matrix remodeling associated 7	protein coding
NFATC4	chr14:24836117-24848811	14q12	nuclear factor of activated T cells 4	protein coding
NLRX1	chr11:119039434-119054725	11q23.3	NLR family member X1	protein coding
PDE1A	chr2:183001180-183291763	2q32.1	phosphodiesterase 1A	protein coding
PRODH	chr22:18900294-18924066	22q11.21	proline dehydrogenase 1	protein coding

RGL1	chr1:183605244-183897677	1q25.3	ral guanine nucleotide dissociation stimulator like 1	protein coding
SEC14L1	chr17:75084767-75213181	17q25.2-q25.3	SEC14 like lipid binding 1	protein coding
SETD1B	chr12:122242630-122270562	12q24.31	SET domain containing 1B, histone lysine methyltransferase	protein coding
SLC13A4	chr7:135365980-135412934	7q33	solute carrier family 13 member 4	protein coding
SLC16A9	chr10:61410522-61469649	10q21.2	solute carrier family 16 member 9	protein coding
SNORA21	chr17:37009116-37009248	17q12	small nucleolar RNA, H/ACA box 21	small nucleolar RNA
SOX5	chr12:23682438-24102604	12p12.1	SRY-box transcription factor 5	protein coding
TC2N	chr14:92246090-92302799	14q32.12	tandem C2 domains, nuclear	protein coding
TCF4	chr18:52889416-53255423	18q21.2	transcription factor 4	protein coding
TGFB3	chr14:76424404-76449354	14q24.3	transforming growth factor beta 3	protein coding
TGM5	chr15:43524788-43559126	5q15.2	transglutaminase 5	protein coding
TLR5	chr1:223282746-223316590	1q41	toll like receptor 5	protein coding
TMEM254	chr10:81838402-81852307	10q22.3	transmembrane protein 254	protein coding
TMTC1	chr12:29653773-29936875	12p11.22	transmembrane O-mannosyltransferase targeting cadherins 1	protein coding
TMX4	chr20:7957993-8000408	20p12.3	thioredoxin related transmembrane protein 4	protein coding
TSPOAP1-AS1	chr17:56414563-56431088	17q22	TSPOAP1, SUPT4H1 and RNF43 antisense RNA 1	protein coding
ZBTB20	chr3:114033364-114866135	3q13.31	zinc finger and BTB domain containing 20	protein coding
ZNF254	chr19:24216207-24312770	19p12	zinc finger protein 254	protein coding
ZNF331	chr19:54024268-54083523	19q13.42	zinc finger protein 331	protein coding
ZNF844	chr19:12175546-12192380	19p13.2	zinc finger protein 844	protein coding
CCDC80	chr3:112315641-112320816	3q13.2	coiled-coil domain containing 80	protein coding

RNF150	chr4:141780961-14254623	4q31.21	ring finger protein 150	protein coding
